# Trajectories of work disability and unemployment before and after a common mental disorder diagnosis among young private sector employees in Sweden–a register-based longitudinal study

**DOI:** 10.1007/s00127-024-02777-0

**Published:** 2024-10-04

**Authors:** Ridwanul Amin, Emma Björkenstam, Magnus Helgesson, Ellenor Mittendorfer-Rutz

**Affiliations:** 1 Division of Insurance Medicine, Department of Clinical Neuroscience, Karolinska Institutet, SE-171 77 Stockholm, Sweden; 2 Division of Infectious Diseases, Department of Medicine, Karolinska Institutet, SE-171 77 Stockholm, Sweden; 3 Department of Public Health and Caring Sciences, Public Health, Working Life and Rehabilitation, Uppsala University, SE-752 37 Uppsala, Sweden

**Keywords:** Sick leave, Disability pension, Cohort, Trajectory

## Abstract

**Purpose:**

To identify trajectory groups of work disability (WD), including sick leave and disability pension, and unemployment three years before and six years (from Y-3 to Y + 6) after a common mental disorder (CMD) diagnosis and to investigate associations of socio-demographic, work-related and clinical factors with trajectory membership.

**Methods:**

A longitudinal nationwide register-based study was conducted including individuals aged 22–29 years, gainfully employed in the private sector, with a CMD diagnosis in specialised healthcare or prescribed antidepressant (N = 12,121) in 2014 (Year 0/Y0), with follow-up from Y-3 to Y + 6. Group-based trajectory analyses identified groups of individuals who followed similar trajectories of months of WD and unemployment, respectively. Multinomial logistic regression determined associations between socio-demographic, work-related and clinical factors and trajectory membership.

**Results:**

In the CMD group, we identified three trajectory groups, each for WD and unemployment. Only 7% individuals belonged to a ‘Fluctuant high’ trajectory group with four months of WD in Y0, which peaked at 7 months in Y + 3 and reduced to 5 months in Y + 6. For unemployment, 15% belonged to an ‘Increasing medium’ trajectory group that steadily increased from 1.3 months in Y0 to 2.6 months in Y + 6. Sex, educational level and musculoskeletal disorders for WD, and educational level, living area and occupational class for unemployment, influentially determined the variance across the CMD trajectory groups.

**Conclusions:**

Specific vulnerable groups regarding unfavourable WD (women, low education and musculoskeletal disorders) and unemployment (manual work, low education and rural residence) trajectories require special attention regarding their return-to-work process following a CMD diagnosis.

**Supplementary Information:**

The online version contains supplementary material available at 10.1007/s00127-024-02777-0.

## Background

Young individuals in many European Union countries, including Sweden, have been struggling to find and keep a job in recent 10–20 years, leading to a rising trend in labour market marginalisation, particularly due to common mental disorders (CMDs) i.e., depressive, anxiety and stress-related mental disorders [1–4]. Although only unemployment is usually considered as a measure of labour market marginalisation, conceptualising it within a social insurance perspective and considering both unemployment and work disability (WD) is more advantageous to capture the full range of adverse consequences of CMDs on unemployment and WD, including sickness absence and disability pension [5]. The rising trend in WD and unemployment among the youth is in line with an increase in the incidence and prevalence of CMDs among adolescents and young adults [6, 7]. CMDs usually have an early age of onset [8], recurrent episodes [9], and they are highly comorbid with other mental and somatic disorders [10]. Therefore, such disorders may jeopardise academic accomplishment and consequently negatively impact the working life through periods of WD and unemployment [11].

A previous study among individuals with a diagnosis of CMDs has shown considerable variations among groups of people who follow specific favourable or unfavourable trajectories of WD and unemployment following a diagnosis of CMD [5]. This study included all individuals 19–30 years of age regardless of being gainfully employed or not. To date, knowledge is lacking about individuals who have established themselves in the labour market through gainful employment who get a diagnosis of CMD. As CMDs usually have an early age of onset, these individuals might already have symptoms of CMD before establishment in the labour market. Moreover, previous research on WD has focused mainly among public sector employees [12–14] who have high levels of WD than those working in the private sector [15–17]. As around 75% of those aged 19–30 years in Sweden work in the private sector [15, 16], exploring WD and unemployment patterns among young private sector employees will contribute to novel findings in an understudied population.

Studies elucidating characteristics of individuals with CMDs with different levels of WD and unemployment should ideally be conceptualised to capture the inherent heterogeneity of individuals with these disorders. Therefore, it is very important to identify different trajectories of WD and unemployment in young employees in the private sector who have CMDs, i.e. showing stable, increasing or decreasing trends of WD and unemployment with low or high levels. Such studies should explore the WD and unemployment patterns not only after the CMD diagnosis but also before the diagnosis in order to identify changes in patterns around the diagnosis.

Moreover, several individual-level factors are associated with WD and unemployment and may differentiate across the identified trajectory groups. Female sex, lower educational level and non-urban residence were found to be associated with higher risk of WD and unemployment [3, 18–21]. Furthermore, levels of WD and unemployment may vary considerably among young employees with or without CMDs across different occupational classes (manual vs. non-manual) and occupational branches (e.g. healthcare) [15, 22]. Moreover, comorbid mental and somatic disorders may further decrease the work capacity and are common among young adults with CMDs [23, 24]. Finding differences in such individual-level factors will help identify specific risk groups. Subsequently, this may help in designing tailor-made interventions to prevent WD and unemployment in this population of young employees in the private sector.

### Aims

The study aimed to identify different trajectory groups of WD and unemployment among young employees in the private sector in Sweden three years before and six years after (from Y-3 to Y + 6) a CMD diagnosis. A further aim was to examine the role of different socio-demographic, work-related and clinical factors in determining the identified trajectories.

## Materials and methods

### Study population

All individuals aged 22–29 years, residents in Sweden on 31-Dec-2014 (N = 1,072,431), were included in the initial study population. This age group (22–29 years) was selected based on the eligibility for being granted temporary disability pension in Sweden (19–29 years) so that the outcome of WD can be measured three years before a CMD diagnosis. Individuals not gainfully employed in 2014 (n = 345,825) or not working in the private sector were then excluded (n = 156,542). Moreover, individuals who had a main diagnosis of CMD in inpatient or specialised outpatient healthcare (mentioned as ‘specialised healthcare’ from here onwards) or had been prescribed any antidepressant medication during 2011–2013 (n = 44,012) were excluded to ensure incident CMD in 2014 among the exposure group in the next step. The International Classification of Diseases version 10 (ICD-10) codes for depressive disorders (F32-33), anxiety disorders (F40-42), and reaction to severe stress and adjustment disorders (F43) or Anatomical Therapeutic Chemical code N06A for any prescribed antidepressant medication use was used to identify CMDs. Of the final study population of 526,052 individuals, n = 12,121 were included as the exposure group with incident CMD in 2014. A 1:1 matched comparison group without incident CMD in 2014 was formed matching on age, sex, educational level and living area. A flowchart (Supplementary Figure S1) shows the selection steps of the study population. For individuals with CMDs, the date of the earliest event of either diagnosis of CMD at specialised healthcare or a prescription of antidepressant in 2014 was considered as the cohort entry date. For the comparison group, the cohort entry date was 1-Jan-2015.

The Swedish health care system is primarily funded by taxpayers and is decentralized. It offers universal access to all residents, with individuals paying limited costs due to tax subsidies. The system is organized around easily accessible primary healthcare centers, where a range of health professionals, including doctors, nurses, and psychologists, work together. Typically, patients first visit a primary healthcare center to see a General Practitioner (GP), who handles most cases. If specialised care is needed, the GP will refer the patient to the appropriate specialist, including mental health professionals. More details of the Swedish healthcare system, including mental healthcare, are provided elsewhere [25, 26]. Based on the findings from a study on all individuals resident in the Stockholm region [27] and the fact that we screened for antidepressant medication use in both primary and specialised healthcare, our study population focusing on specialised healthcare can be estimated to include around half of the study population of individuals with similar inclusion criteria who sought mental healthcare for CMDs in any level of healthcare (primary or specialised) in 2014.

### Data sources

We identified the study population by linking information from three nationwide Swedish registers: (1) Longitudinal Integration Database for Health Insurance and Labor Market Studies (LISA) register [28], held by Statistic Sweden; (2) National Patient Register [29, 30] and (3) Prescribed Drug Register [31], both maintained by the National Board of Health and Welfare. Data on age, sex, educational level, living area, family situation, emigration, employment sector, occupational class and unemployment were extracted from LISA. Information on specialised healthcare for CMDs and other comorbidities (clinical factors) was identified from the National Patient Register. The Prescribed Drug Register was used to obtain data on prescribed antidepressants. Information regarding age at onset, duration and underlying diagnosis of sickness absence and receipt of disability pension was collected from the Micro-Data for Analysis of the Social Insurance System [32] database, held by the Swedish Social Insurance Agency. The Cause of Death Register [33], maintained by the National Board of Health and Welfare, was used to obtain death-related information. Data from the above registers were linked using pseudonymised unique personal identity numbers [28] assigned to all individuals born or registered to be living in Sweden.

### Outcome measures

In Sweden, individuals ≥ 16 years old, with previous employment (or an expected work) lasting at least six months, are entitled to sickness absence benefits if their work capacity is reduced by at least 25% due to disease or injury [28]. After one qualifying day, the next 14 days of sickness absence benefit is paid by the employer, and after that, sickness absence benefits are compensated by the Social Insurance Agency [28]. According to the reduction of work capacity, sickness absence benefits can be claimed full- or part-time (i.e. 25%, 50% or 75%). All individuals 30–64 years of age with permanent impairment of work capacity due to disease or injury can be granted permanent disability pension [28]. Temporary disability pension can be granted to individuals 19–29 years of age with reduced work capacity which is expected to continue for a minimum of one year or who have not completed their education due to health issues [28].

In this study, WD was defined as annual net days of sickness absence or disability pension during 2011–2020, i.e. three years before, during and six years after the year of the cohort entry date. The follow-up period was chosen according to data availability, age-eligibility criteria for the outcome of WD and methods used in previous literature [5]. The observation years were calendar years instead of periods of 365 days before and after the cohort entry date. The net days were calculated by considering the extent of the sickness absence/disability pension spell (25%-75%).

Unemployment insurance includes basic as well as income-related optional insurance in Sweden. Individuals aged 20–64 years without voluntary income-related insurance are eligible for basic unemployment benefit given that they fulfil certain prerequisites: being registered at the National Public Employment Service as a job seeker and being ready to enter the labour market if a suitable job is offered [28]. Unemployment benefits cover up to 80% of the income lost; with an upper threshold of 910 SEK/day during the first 200 days post-unemployment and then 70% until day 300 [28]. Unemployment was defined as annual days of being enrolled as full-time unemployed at the Swedish Public Employment Service from 2011 and 2020.

### Covariates

A. Socio-demographic factors (sex, age, educational level, family situation and living area); B. Work-related factors (occupational class, occupational branch); C. Clinical factors (history of any specialised healthcare use due to specific mental or somatic disorders (ICD-10 codes)—substance use disorders (F10-19), Attention deficit hyperactivity disorder (F90), other mental disorders (other F codes except F32-33, F40-43), diabetes mellitus (E10-14), asthma (J45), cardiovascular disorders (I00-99), neoplasm (C00-D49), musculoskeletal disorders (M00-99), other disorders (other ICD-10 codes)). Socio-demographic and work-related factors were measured on 31-Dec of the year before cohort entry date. Clinical factors were measured during the three years preceding cohort entry date. Individuals with missing data for any variable were categorised as a separate group. The categories for all covariates are shown in Table 1.

**Table 1:** Distribution of the socio-demographic, work-related and clinical factors among young employees in the private sector with^a^ (n = 12,121) or without^a^ (1:1 matched on age, sex, educational level and living area) an incident diagnosis of common mental disorders (CMD^b^) during 2014, aged 22–29 years, gainfully employed and residing in Sweden on 31-Dec 2014

Characteristics	With incident CMD n (column %)	Without incident CMD n (column %)	Chi-square (χ2) test p-value
*Socio-demographic factors*^*c*^			
Sex			
Men	5452 (45.0)	5452 (45.0)	NA^g^
Women	6669 (55.0)	6669 (55.0)	
Age (years)			
22–24	4461 (36.8)	4461 (36.8)	NA^g^
25–29	7660 (63.2)	7660 (63.2)	
Educational level (years)			
Compulsory school (0–9)	1403 (11.6)	1403 (11.6)	NA^g^
High school (10–12)	7157 (59.0)	7157 (59.0)	
College or university (> 12)	3481 (28.7)	3481 (28.7)	
Missing	80 (0.7)	80 (0.7)	
Family situation			
Single	10,163 (83.8)	9543 (78.7)	< 0.001
Married/cohabiting	1958 (16.2)	2578 (21.3)	
Living area^d^			
Small cities/villages	2595 (21.4)	2595 (21.4)	NA^g^
Medium-sized cities	4009 (33.1)	4009 (33.1)	
Big cities	5517 (45.5)	5517 (45.5)	
*Work-related factors*^*c*^			
Occupational class			
Manual worker	4615 (38.1)	4868 (40.2)	< 0.001
Non-manual worker	1590 (13.1)	1835 (15.1)	
Missing	5916 (48.8)	5418 (44.7)	
Occupational branch			
Agriculture	110 (0.9)	183 (1.5)	< 0.001
Commerce and transport	4261 (35.2)	4587 (37.8)	
Construction	942 (7.8)	1086 (9.0)	
Education and research	364 (3.0)	363 (3.0)	
Financial and enterprise	403 (3.3)	454 (3.7)	
Healthcare	1134 (9.4)	868 (7.2)	
Industry	1365 (11.3)	1368 (11.3)	
Others	3542 (29.2)	3212 (26.5)	
*Clinical factors*^*c*^* (ICD-10 codes)*			
History of healthcare use for mental disorders^e^ (Yes)			
Substance use disorders (F10-19)	348 (2.9)	134 (1.1)	< 0.001
Attention deficit hyperactivity disorder (F90)	239 (2.0)	86 (0.7)	< 0.001
Other disorders (other F codes except F32-33, F40-43)	332 (2.7)	100 (0.8)	< 0.001
History of healthcare use for somatic disorders^f^ (Yes)			
Diabetes mellitus (E10-14)	101 (0.8)	76 (0.6)	0.059
Asthma (J45)	172 (1.4)	117 (1.0)	0.001
Cardiovascular disorders (I00-99)	257 (2.1)	187 (1.5)	< 0.001
Neoplasm (C00-D49)	528 (4.4)	439 (3.6)	0.003
Musculoskeletal disorders (M00-99)	1407 (11.6)	918 (7.6)	< 0.001
Other disorders (other ICD-10 codes)	8140 (67.2)	6528 (53.9)	< 0.001

^a^Individuals with or without incident CMD as the exposure and comparison group, respectively

^b^Any main diagnosis of CMD at inpatient or specialised outpatient healthcare or any prescription of antidepressant medication during 2014. The following codes were used to identify incident CMD: the International Classification of Diseases version 10 (ICD-10) codes for depressive disorders (F32-33), anxiety disorders (F40-42), and reaction to severe stress and adjustment disorders (F43) or Anatomical Therapeutic Chemical (ATC) code N06A for any prescribed antidepressant medication use

^c^All socio-demographic and work-related factors were measured at 31-Dec of the year before cohort entry date. The clinical factors were measured for a three-year period before the cohort entry

^d^Living area: big cities—Stockholm, Gothenburg and, Malmö; medium-sized cities—cities with more than 90,000 inhabitants within 30 km distance from the centre of the city; small cities/villages

^e^Measured as any inpatient or specialised outpatient healthcare due to specific mental disorders diagnosis except CMDs (any ICD-10 F code except F32-33 and F40-43 codes). ‘No history of specialised healthcare use for a specific mental disorder diagnoses’ category is not presented

^f^Measured as any inpatient or specialised outpatient healthcare due to a specific somatic diagnosis (any ICD-10 code except ‘F’, ‘O’, ‘P’ and ‘Q’ codes). ‘No history of specialised healthcare use for specific somatic diagnoses’ category is not presented

^g^Not applicable for a matching variable

### Statistical analysis

Differences in the distributions of the covariates between the exposure and comparison groups were tested using the Chi-square (χ2) test. Group-based trajectory modelling [34, 35] was used to estimate trajectory groups of WD and unemployment among young employees in the private sector during 2011–2020, i.e. three years before (Y-3) to six years after (Y + 6) the year of cohort entry (Y0). Group-based trajectory modelling considers the trajectory groups as an approximation of the heterogeneity within a population. It does not follow the assumption of latent growth mixture modelling, which states that there are distinct subpopulations within the population [36]. Moreover, residual variance is considered to be constant across classes and time points [37]. We preferred this method as it is computationally less demanding and has fewer issues with model convergence. Bayesian information criterion (BIC) was used to identify the best-fitted model. In addition to BIC values, a requirement of at least 5% of the study population in each of the subgroups was introduced for model selection to ensure adequate statistical power in the subsequent logistic regression analysis. Probability of an individual belonging to a specific trajectory group was calculated and the highest estimated probability was considered for group belonging. Here, an average probability of ≥ 0.70 for individuals of a trajectory group, as suggested by Cote and co-authors, was used as an indication for the goodness of fit [34]. We followed the guidelines for reporting on latent trajectory studies [38] and presented a checklist and relevant information in Supplementary information 1, 2.

To explore how much of the variability of the identified trajectory groups is explained by different individual-level characteristics, we used multinomial logistic regression to test the associations among the trajectory groups and all the covariates. Log-likelihood χ2 tests were applied to estimate if the covariates were associated with a specific trajectory group. Nagelkerke pseudo-R^2^ values were applied to evaluate the strength of such potential associations by comparing the full model with the model without the specific covariate. In case of death or emigration during a specific year of follow-up, an individual’s outcome data was considered missing for that year and the subsequent follow-up years. For all analyses, a p-value < 0.05 was considered statistically significant. Data analysis was performed using SAS v. 9.4 (“Proc Traj” was used for the group-based trajectory analysis) [35].

### Sensitivity analyses

As the distributions of some of the covariates changed over time, in a sensitivity analysis, we included educational level, healthcare use for other mental disorders than CMD and healthcare use for somatic disorders as time-varying in the trajectory models. The risk of WD and unemployment is higher among individuals with psychiatric morbidity. Therefore, in another sensitivity analysis, we included only those without a record of specialised healthcare with any ICD-10 F codes during 2011–2014 (n = 7498 in the exposure group and n = 11,802 in the comparison group).

## Results

Table 1 shows the distributions of the different covariates among the CMD group and the comparison group. A lower proportion of individuals with incident CMD at cohort entry (16%) were married/cohabiting than the non-CMD comparison group (21%) (χ2 test p-value < 0.001). Fewer individuals were working in commerce and transport (35% vs 38%) and construction (8% vs 9%), and more individuals were working in healthcare (9% vs 7%) among the CMD group. History of healthcare use for substance use disorders, attention deficit hyperactivity disorders and other mental disorders except CMDs were around 2% higher among the CMD group than the comparison group (Table 1, χ2 test p-value < 0.001). History of healthcare use for asthma (1.4% vs 1%), cardiovascular disorders (2.1% vs 1.5%), neoplasm (4.4% vs 3.6%), musculoskeletal disorders (12% vs 8%), and other somatic disorders were statistically significantly higher (χ2 test p-value < 0.01) among the CMD group than the comparison group.

### Trajectory groups for work disability

**Fig. 1:**
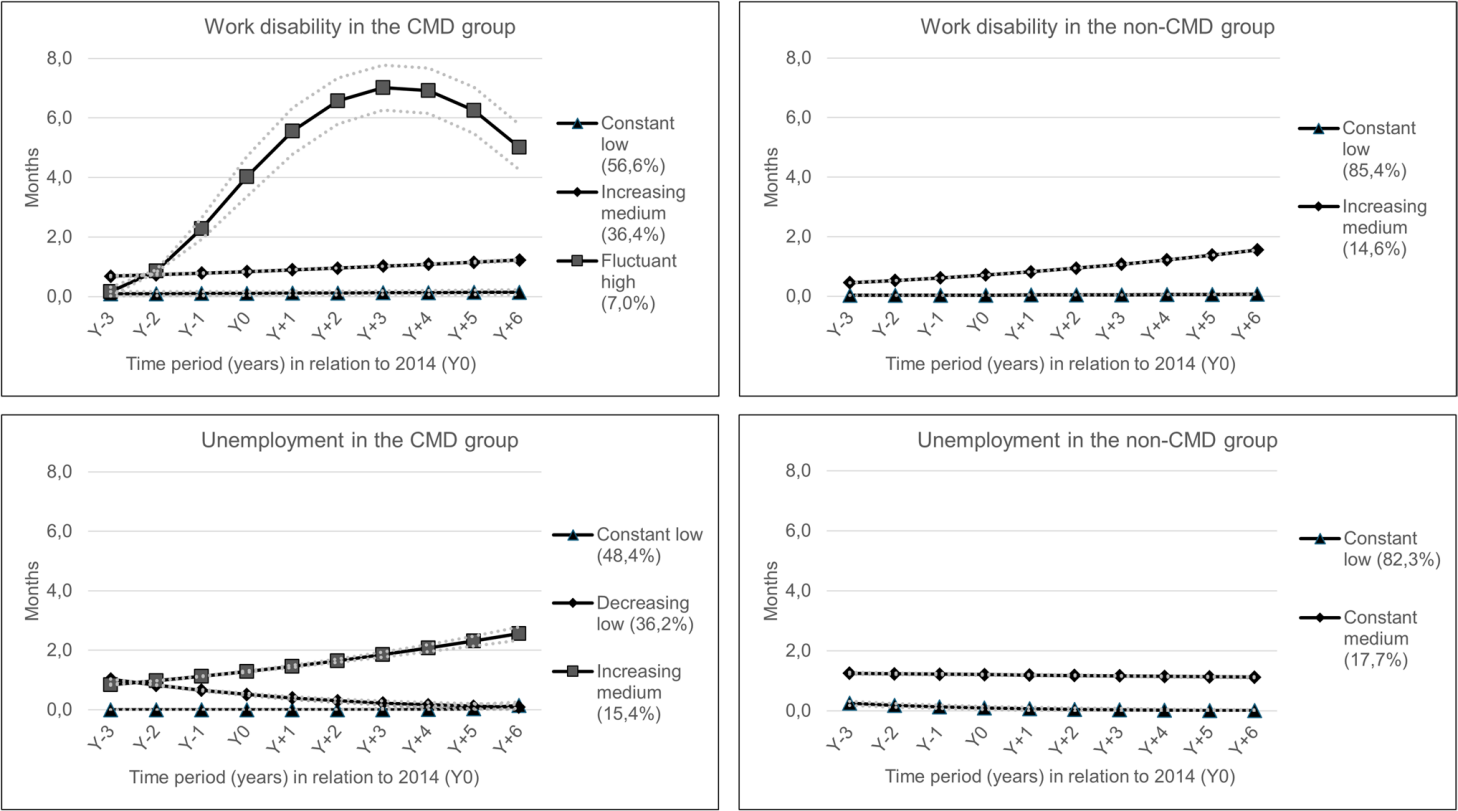
Trajectory groups of work disability and unemployment three years before (Y-3) and six years after (Y + 6) an incident (Y0) diagnosis of common mental disorder (CMD) in 2014 among young employees in the private sector, aged 22–29 years, gainfully employed and residing in Sweden on 31-Dec 2014 (n = 12,121; exposure group) and the 1:1 matched (on age, sex, educational level and living area) comparison group without any incident CMD in 2014

We identified three trajectory groups (‘Constant low’, ‘Increasing medium’ and ‘Fluctuant high’) among the CMD group and two trajectory groups (‘Constant low’, ‘Increasing medium’) among the non-CMD comparison group (Fig. 1). Most individuals in both CMD (57%) and non-CMD groups (85%) belonged to the ‘Constant low’ trajectory groups and had 0.1 months of WD throughout the observation period. A gradual increase of months of WD was observed among the ‘Increasing medium’ groups, from 0.5 and 0.7 months in Y-3 to 1.3–1.6 months in Y + 6 for the CMD group (36%) and non-CMD group (15%). The ‘Fluctuant high’ (7%) group had 0.2 months of WD in Y-3, which increased to four months at Y0, peaked at seven months in Y + 3 and then decreased to five months in Y + 6.

The distributions and associations of socio-demographic, work-related and clinical factors across the identified trajectory groups among the CMD and non-CMD groups are presented in Table 2. The full model, including all covariates, explained 12% of the variance across these trajectory groups (Nagelkerke pseudo-R^2^ value 0.121) for the CMD group and 9% for the non-CMD group (Nagelkerke pseudo-R^2^ value 0.088). Sex, educational level and history of healthcare use for musculoskeletal disorders explained 2%, 3% and 1% of the variance as influential individual factors (difference in pseudo-R^2^ = 0.016, 0.032 and 0.011, respectively) for the CMD group. For the non-CMD group, sex, educational level and history of healthcare use for other somatic disorders explained 2%, 1% and 2% of the variance as influential factors (difference in pseudo-R^2^ = 0.015, 0.014 and 0.016, respectively). Other covariates had little to no effect in explaining the variance in the full model (difference in pseudo-R^2^ =  ≤ 0.01; Table 2) for both CMD and non-CMD groups.

**Table 2:** Distributions and associations of socio-demographic, work-related and clinical characteristics in each trajectory group of work disability^a^ among the exposure group of young employees in the private sector with an incident diagnosis of common mental disorders (CMD^b^) during 2014, aged 22–29 years, gainfully employed and residing in Sweden on 31-Dec 2014 (n = 12,121), and among their 1:1 matched^c^ comparison group

Characteristics	CMD group					Non-CMD comparison group			
	Constant low n (column %)	Increasing medium n (column %)	Fluctuant high n (column %)	p-value of log-likelihood χ2 test	Difference^gh^ in Nagelkerke pseudo-R^2^	Constant low n (column %)	Increasing medium n (column %)	p-value of log-likelihood χ^2^ test	Difference^h^ in Nagelkerke pseudo-R^2^
All (row percentage)	4414 (36.4)	6856 (56.6)	851 (7.0)			10,348 (85.4)	1773 (14.6)		
*Socio-demographic factors*^*d*^									
Sex									
Men	3408 (49.7)	1703 (38.6)	341 (40.1)	< 0.001	0.016	4854 (46.9)	598 (33.7)	< 0.001	0.015
Women	3448 (50.3)	2711 (61.4)	510 (59.9)			5494 (53.1)	1175 (66.3)		
Age (years)									
22–24	2654 (38.7)	1528 (34.6)	279 (32.8)	< 0.001	0.004	3845 (37.2)	616 (34.7)	0.001	0.001
25–29	4202 (61.3)	2886 (65.4)	572 (67.2)			6503 (62.8)	1157 (65.3)		
Educational level (years)									
Compulsory school (0–9)	618 (09.0)	620 (14.0)	165 (19.4)	< 0.001	0.032	1099 (10.6)	304 (17.1)	< 0.001	0.014
High school (10–12)	3792 (55.3)	2808 (63.6)	557 (65.5)			6036 (58.3)	1121 (63.2)		
College/university (> 12)	2397 (35.0)	961 (21.8)	123 (14.5)			3142 (30.4)	339 (19.1)		
Missing	49 (0.7)	25 (0.6)	6 (0.7)			71 (0.7)	9 (0.5)		
Family situation									
Single	6002 (87.5)	3476 (78.7)	685 (80.5)	< 0.001	0.006	8246 (79.7)	1297 (73.2)	0.033	0
Married/cohabiting	854 (12.5)	938 (21.3)	166 (19.5)			2102 (20.3)	476 (26.8)		
Living area^e^									
Small cities/villages	1328 (19.4)	1042 (23.6)	225 (26.4)	< 0.001	0.002	2177 (21.0)	418 (23.6)	0.504	0
Medium-sized cities	2223 (32.4)	1485 (33.6)	301 (35.4)			3435 (33.2)	574 (32.4)		
Big cities	3305 (48.2)	1887 (42.8)	325 (38.2)			4736 (45.8)	781 (44.0)		
*Work-related factors*^*d*^									
Occupational class									
Manual worker	2596 (37.9)	1728 (39.1)	291 (34.2)	< 0.001	0.003	4115 (39.8)	753 (42.5)	0.022	0.001
Non-manual worker	1010 (14.7)	517 (11.7)	63 (7.4)			1648 (15.9)	187 (10.5)		
Missing	3250 (47.4)	2169 (49.1)	497 (58.4)			4585 (44.3)	833 (47.0)		
Occupational branch									
Agriculture	69 (1.0)	30 (0.7)	11 (1.3)	< 0.001	0.005	162 (1.6)	21 (1.2)	0.015	0.002
Commerce and transport	2468 (36.0)	1516 (34.3)	277 (32.5)			3923 (37.9)	664 (37.5)		
Construction	525 (7.7)	370 (8.4)	47 (5.5)			929 (9.0)	157 (8.9)		
Education and research	176 (2.6)	161 (3.6)	27 (3.2)			295 (2.9)	68 (3.8)		
Financial and enterprise	253 (3.7)	126 (2.9)	24 (2.8)			401 (3.9)	53 (3.0)		
Healthcare	562 (8.2)	481 (10.9)	91 (10.7)			704 (6.8)	164 (9.2)		
Industry	757 (11.0)	502 (11.4)	106 (12.5)			1164 (11.2)	204 (11.5)		
Others	2046 (29.8)	1228 (27.8)	268 (31.5)			2770 (26.8)	442 (24.9)		
*Clinical factors*^*d*^									
History of healthcare use for mental disorders^f^ (Yes)									
Substance use disorders	159 (2.3)	155 (3.5)	34 (4.0)	0.32	0.001	103 (1.0)	31 (1.7)	0.198	0
ADHD	97 (1.4)	106 (2.4)	36 (4.2)	0.19	0.001	58 (0.6)	28 (1.6)	0.013	0.001
Other mental disorders	126 (1.8)	146 (3.3)	60 (7.1)	< 0.001	0.004	59 (0.6)	41 (2.3)	< 0.001	0.003
History of healthcare use for somatic disorders^g^ (Yes)									
Diabetes mellitus	40 (0.6)	49 (1.1)	12 (1.4)	0.012	0.001	65 (0.6)	11 (0.6)	0.467	0
Asthma	67 (1.0)	93 (2.1)	12 (1.4)	0.003	0.001	91 (0.9)	26 (1.5)	0.232	0
Cardiovascular disorders	115 (1.7)	101 (2.3)	41 (4.8)	< 0.001	0.002	143 (1.4)	44 (2.5)	0.084	0
Neoplasm	262 (3.8)	208 (4.7)	58 (6.8)	0.34	0.001	348 (3.4)	91 (5.1)	0.112	0
Musculoskeletal disorders	562 (8.2)	697 (15.8)	148 (17.4)	< 0.001	0.011	673 (6.5)	245 (13.8)	< 0.001	0.008
Other somatic disorders	4226 (61.6)	3290 (74.5)	624 (73.3)	< 0.001	0.006	5273 (51.0)	1255 (70.8)	< 0.001	0.016

^a^Work disability measured as annual net days due to sickness absence or disability pension. The net days were calculated by multiplying days spent on part-time sickness absence/disability pension by the extent (25%-75%)

^b^Any main diagnosis of CMD at inpatient or specialised outpatient healthcare or any prescription of antidepressant medication during 2014. The following codes were used to identify incident CMD: the International Classification of Diseases version 10 (ICD-10) codes for depressive disorders (F32-33), anxiety disorders (F40-42), and reaction to severe stress and adjustment disorders (F43) or Anatomical Therapeutic Chemical (ATC) code N06A for any prescribed antidepressant medication use

^c^Matching factors: age, sex, educational level and living area

^d^All socio-demographic and work-related factors were measured at 31-Dec of the year before cohort entry date. The clinical factors were measured for a three-year period before the cohort entry

^e^Living area: big cities—Stockholm, Gothenburg and, Malmö; medium-sized cities—cities with more than 90,000 inhabitants within 30 km distance from the centre of the city; small cities/villages

^f^Measured as any inpatient or specialised outpatient healthcare due to any mental disorder diagnosis except CMDs (any ICD-10 F code except F32-33 and F40-43 codes). ‘No history of specialised healthcare use for a mental disorder diagnoses’ category is not presented. Substance use disorders (F10-19), Attention Deficit Hyperactivity disorder/ADHD (F90), other mental disorder (other F codes except F32-33 and F40-43)

^g^Measured as any inpatient or specialised outpatient healthcare due to any somatic diagnosis (any ICD-10 code except ‘F’, ‘O’, ‘P’ and ‘Q’ codes). ‘No history of specialised healthcare use for somatic diagnoses’ category is not presented. Diabetes mellitus (E10-14), Asthma (J45), Cardiovascular disorders (I00-99), Neoplasms (C00-D49), Musculoskeletal disorders (M00-99), other somatic disorders (other ICD-10 codes)

^h^Difference in Nagelkerke pseudo-R^2^ between model including tested variable and model without tested variable. Nagelkerke pseudo-R^2^ for full model, including all socio-demographic, work-related and clinical factors for the CMD and non-CMD groups are 0.121 and 0.088, respectively

The ‘Constant low’ trajectory group among those with CMD had a higher proportion of individuals with a history of other somatic disorders (62%) than the ‘Constant low’ non-CMD group (51%). The distribution for other covariates between the ‘Constant low’ CMD and non-CMD group and the distribution for all covariates between the ‘Increasing medium’ CMD and non-CMD groups were similar (Table 2). Comparison among the three trajectory groups within those with CMD revealed that both the ‘Increasing medium’ (61%) and the ‘Fluctuant high’ (60%) groups had a higher proportion of women than the ‘Constant low’ group (50%). The ‘Fluctuant high’ group was less likely to have individuals with college/university education (15% vs 22%-35%) than the other trajectory groups among those with CMD. This group was also more likely to have individuals with musculoskeletal (17% vs 8%) or other somatic disorders (73% vs 62%) than the ‘Constant low’ group.

### Trajectory groups for unemployment

The three identified trajectory groups among the CMD group were ‘Constant low’, ‘Decreasing low’ and ‘Increasing medium’, and among the non-CMD group, the two identified trajectory groups were ‘Constant low’ and ‘Constant medium’ (Fig. 1). For both the CMD (48%) and non-CMD groups (82%), ‘Constant low’ trajectory constituted the largest group with 0–0.1 months of unemployment between Y-3 and Y + 6. The ‘Decreasing low’ CMD trajectory group (36%) had one month of unemployment at Y-3, which gradually declined to 0.1 months at Y + 6. The ‘Increasing medium’ CMD group followed the opposite trajectory, from 0.8 months of unemployment at Y-3, gradually increasing to 2.6 months at Y + 6. The ‘Constant medium’ non-CMD trajectory group had around 1.2 months of unemployment throughout the observation period (Fig. 1).

Table 3 shows the distributions and associations of socio-demographic, work-related and clinical factors across the identified trajectory groups among the CMD and the non-CMD groups, respectively. The full model, including all covariates, explained 11% of the variance across the CMD trajectory groups (Nagelkerke pseudo-R^2^ value 0.109) and 8% for the non-CMD trajectory groups (Nagelkerke pseudo-R^2^ value 0.077). Educational level, living area and occupational class explained 3%, 1% and 1% of the variance as individual factors (difference in pseudo-R^2^ = 0.027, 0.013 and 0.014, respectively) for the CMD group. For the non-CMD group, only educational level explained 3% of the variance as an influential individual factor (difference in pseudo-R^2^ = 0.032). Other covariates had little to no contribution in explaining the variance of the full model (difference in pseudo-R^2^ =  ≤ 0.01; Table 3) for both CMD and non-CMD groups.

**Table 3:** Distributions and associations of socio-demographic, work-related and clinical characteristics in each trajectory group of unemployment^a^ among the exposure group of young employees in the private sector with an incident diagnosis of common mental disorders (CMD^b^) during 2014, aged 22–29 years, gainfully employed and residing in Sweden on 31-Dec 2014 (n = 12,121), and among their 1:1 matched^c^ comparison group

Characteristics	CMD group					Non-CMD comparison group			
	Constant low n (column %)	Decreasing low n (column %)	Increasing medium n (column %)	p-value of log-likelihood χ2 test	Difference^gh^ in Nagelkerke pseudo-R^2^	Constant low n (column %)	Constant medium n (column %)	p-value of log-likelihood χ^2^ test	Difference^h^ in Nagelkerke pseudo-R^2^
All (row percentage)	5868 (48.4)	4389 (36.2)	1864 (5.0)			9977 (82.3)	2144 (17.7)		
*Socio-demographic factors*^*d*^									
Sex									
Men	2550 (43.5)	1928 (43.9)	974 (52.3)	< 0.001	0.002	4405 (44.2)	1047 (48.8)	0.009	0.001
Women	3318 (56.5)	2461 (56.1)	890 (47.7)			5572 (55.8)	1097 (51.2)		
Age (years)									
22–24	1810 (30.8)	1941 (44.2)	710 (38.1)	< 0.001	0.008	3577 (35.9)	884 (41.2)	0.042	0
25–29	4058 (69.2)	2448 (55.8)	1154 (61.9)			6400 (64.1)	1260 (58.8)		
Educational level (years)									
Compulsory school (0–9)	466 (7.0)	542 (12.3)	395 (21.2)	< 0.001	0.027	913 (9.2)	490 (22.9)	< 0.001	0.032
High school (10–12)	3200 (54.5)	2831 (64.5)	1126 (60.4)			5894 (59.1)	1263 (58.9)		
College/university (> 12)	2162 (36.8)	997 (22.7)	322 (17.3)			3106 (31.1)	375 (17.5)		
Missing	40 (0.7)	19 (0.4)	21 (1.1)			64 (0.6)	16 (0.7)		
Family situation									
Single	4850 (82.7)	3735 (85.1)	1578 (84.7)	< 0.001	0.002	7854 (78.7)	1689 (78.8)	0.252	0
Married/cohabiting	1018 (17.3)	654 (14.9)	286 (15.3)			2123 (21.3)	455 (21.2)		
Living area^e^									
Small cities/villages	1028 (17.5)	1120 (25.5)	447 (24.0)	< 0.001	0.013	2073 (20.8)	522 (24.3)	0.002	0.001
Medium-sized cities	1770 (30.2)	1580 (36.0)	659 (35.4)			3257 (32.6)	752 (35.1)		
Big cities	3070 (52.3)	1689 (38.5)	758 (40.7)			4647 (46.6)	870 (40.6)		
*Work-related factors*^*d*^									
Occupational class									
Manual worker	2197 (37.4)	1768 (40.3)	650 (34.9)	< 0.001	0.014	4008 (40.2)	860 (40.1)	< 0.001	0
Non-manual worker	1072 (18.3)	377 (8.6)	141 (7.6)			1688 (16.9)	147 (6.9)		
Missing	2599 (44.3)	2244 (51.1)	1073 (57.6)			4281 (42.9)	1137 (53.0)		
Occupational branch									
Agriculture	56 (1.0)	41 (0.9)	13 (0.7)	< 0.001	0.01	156 (1.6)	27 (1.3)	< 0.001	0.007
Commerce and transport	2064 (35.2)	1531 (34.9)	666 (35.7)			3748 (37.6)	839 (39.1)		
Construction	490 (8.4)	318 (7.2)	134 (7.2)			922 (9.2)	164 (7.6)		
Education and research	193 (3.3)	122 (2.8)	49 (2.6)			307 (3.1)	56 (2.6)		
Financial and enterprise	246 (4.2)	117 (2.7)	40 (2.1)			392 (3.9)	62 (2.9)		
Healthcare	554 (9.4)	436 (9.9)	144 (7.7)			693 (6.9)	175 (8.2)		
Industry	655 (11.2)	490 (11.2)	220 (11.8)			1,158 (11.6)	210 (9.8)		
Others	1610 (27.4)	1334 (30.4)	598 (32.1)			2601 (26.1)	611 (28.5)		
*Clinical factors*^*d*^									
History of healthcare use for mental disorders^f^ (Yes)									
Substance use disorders	126 (2.1)	124 (2.8)	98 (5.3)	< 0.001	0.002	95 (1.0)	39 (1.8)	0.096	0
ADHD	61 (1.0)	99 (2.3)	79 (4.2)	< 0.001	0.003	42 (0.4)	44 (2.1)	< 0.001	0.004
Other mental disorders	116 (2.0)	143 (3.3)	73 (3.9)	0.001	0.001	70 (0.7)	30 (1.4)	0.436	0
History of healthcare use for somatic disorders^g^ (Yes)									
Diabetes mellitus	44 (0.7)	39 (0.9)	18 (1.0)	0.991	0	67 (0.7)	9 (0.4)	0.169	0
Asthma	74 (1.3)	71 (1.6)	27 (1.4)	0.264	0	99 (1.0)	18 (0.8)	0.46	0
Cardiovascular disorders	119 (2.0)	100 (2.3)	38 (2.0)	0.644	0	151 (1.5)	36 (1.7)	0.605	0
Neoplasm	286 (4.9)	166 (3.8)	76 (4.1)	0.085	0.001	374 (3.7)	65 (3.0)	0.233	0
Musculoskeletal disorders	652 (11.1)	534 (12.2)	221 (11.9)	0.684	0	757 (7.6)	161 (7.5)	0.404	0
Other somatic disorders	3871 (66.0)	2996 (68.3)	1273 (68.3)	0.117	0.001	5331 (53.4)	1197 (55.8)	0.398	0

^a^Annual days enrolled as full-time unemployed at the Swedish Public Employment Service

^b^Any main diagnosis of CMD at inpatient or specialised outpatient healthcare or any prescription of antidepressant medication during 2014. The following codes were used to identify incident CMD: the International Classification of Diseases version 10 (ICD-10) codes for depressive disorders (F32-33), anxiety disorders (F40-42), and reaction to severe stress and adjustment disorders (F43) or Anatomical Therapeutic Chemical (ATC) code N06A for any prescribed antidepressant medication use

^c^Matching factors: age, sex, educational level and living area

^d^All socio-demographic and work-related factors were measured at 31-Dec of the year before cohort entry date. The clinical factors were measured for a three-year period before the cohort entry

^e^Living area: big cities—Stockholm, Gothenburg and, Malmö; medium-sized cities—cities with more than 90,000 inhabitants within 30 km distance from the centre of the city; small cities/villages

^f^Measured as any inpatient or specialised outpatient healthcare due to any mental disorder diagnosis except CMDs (any ICD-10 F code except F32-33 and F40-43 codes). ‘No history of specialised healthcare use for a mental disorder diagnoses’ category is not presented. Substance use disorders (F10-19), Attention Deficit Hyperactivity disorder/ADHD (F90), other mental disorder (other F codes except F32-33 and F40-43)

^g^Measured as any inpatient or specialised outpatient healthcare due to any somatic diagnosis (any ICD-10 code except ‘F’, ‘O’, ‘P’ and ‘Q’ codes). ‘No history of specialised healthcare use for somatic diagnoses’ category is not presented. Diabetes mellitus (E10-14), Asthma (J45), Cardiovascular disorders (I00-99), Neoplasms (C00-D49), Musculoskeletal disorders (M00-99), other somatic disorders (other ICD-10 codes)

^h^Difference in Nagelkerke pseudo-R^2^ between model including tested variable and model without tested variable. Nagelkerke pseudo-R^2^ for full model, including all socio-demographic, work-related and clinical factors for the CMD and non-CMD groups are 0.109 and 0.077, respectively

Compared with the ‘Constant low’ non-CMD group, a higher proportion of individuals had college/university education (31% vs 37%) and lived in big cities (47% vs 52%) among the ‘Constant low’ CMD group. Those in the ‘Decreasing low’ CMD group were more likely to have a higher educational level (23%) than both the ‘Increasing medium’ CMD group (17%) and ‘Constant medium’ non-CMD group (17%).

### Sensitivity analysis

The identified trajectory groups (Supplementary Fig. 2a–c) in the models adjusting for time-varying covariates (educational level, healthcare use for other mental disorders than CMD or somatic disorders), and the models excluding individuals with specialised healthcare with any ICD-10 F codes during 2011–2014 (Supplementary Fig. 3), were similar to the results from our main analysis.

## Discussion

### Main findings

We identified three trajectories, each for the outcome of WD and unemployment, during three years before and six years after an incident CMD diagnosis among 12,121 private sector employees aged 22–29 years. For the comparison group without an incident CMD diagnosis, two trajectories were identified for each outcome. Only 7% of in the CMD group belonged to a ‘Fluctuant high’ trajectory group with four months of WD in Y0, which peaked at seven months in Y + 3 and reduced to five months in Y + 6. The ‘Constant low’ (85%) and ‘Increasing medium’ (15%) trajectory groups of WD in the comparison group had similar months of WD during the follow-up period as that for the CMD group. For unemployment among the CMD group, 15% belonged to an ‘Increasing medium’ trajectory group that steadily increased from 1.3 months in Y0 to 2.6 months in Y + 6. The rest of the CMD group belonged to ‘Constant low’ (48%, 0 months between Y-3 and Y + 6) and ‘Decreasing low’ (36%, one month in Y-3 to 0.1 months in Y + 6) trajectory groups. The non-CMD comparison group had two constant trajectories of unemployment at low (82%, 0.1 months on average between Y-3 and Y + 6) and medium (18%, 1.2 months on average between Y-3 and Y + 6) levels. In the CMD group, sex, educational level and musculoskeletal disorders for WD, and educational level, living area and occupational class for unemployment, influentially determined the variance across the trajectory groups.

### Discussion of the results

A previous study on trajectories of WD and unemployment before and after an incident diagnosis of CMD among youth aged 19–30 years identified five different trajectories for each outcome among both the CMD group and their non-CMD comparison group [5]. However, they included a more general study population, irrespective of their sector of employment or history of prior labour market attachment. The trajectory membership among young private sector employees in our study followed more homogenous patterns (three trajectories in the CMD group and two trajectories in the comparison group for both WD and unemployment) than that study. Individuals working in the private sector with previous attachment to the labour market through gainful employment can be considered to have lower variability in within-group characteristics than in a group not differentiated by employment sector or history of employment. Inclusion of only private sector employees with gainful employment at baseline leading to selection of a more homogenous group in our study may partly explain these differences. Moreover, the age range in the current study is narrower than the previous study. Alaie et al. 2023 [39] included 669 private sector employees aged 19–20 years who had elevated depressive/anxiety symptoms in Swedish surveys completed in 2005 and followed them for the outcome of annual net days of SA, spanning from 2006 to 2018 (13 years). They identified four latent trajectories labelled: ‘Medium-increasing’ (6%), ‘High-decreasing’ (7%), ‘Medium-fluctuant’ (14%), and ‘Low-constant’ (72%). A lower proportion (57%) of individuals belonging to the ‘Constant low’ trajectory in our study could be due to the differences in study populations and outcome measurements. The youth in this study with physician-diagnosed CMDs might have had a higher level of disease burden and subsequent WD than the youth who self-reported elevated depressive/anxiety symptoms. Moreover, we included days with DP in the outcome. Due to the introduction of much stricter regulations in 2008 for being granted DP in Sweden, DP rates declined considerably in the later cohorts, in general [3] and specifically among young private sector employees [15]. This may suggest a higher level of severity of CMDs among those being granted DP than those receiving SA benefits only.

The majority of individuals in the CMD group followed ‘Constant low’ WD and unemployment trajectories during the follow-up period (57% and 48%, respectively). However, these proportions were much lower than those in the non-CMD group (85% and 82%, respectively). These findings show that youth in the private sector with CMDs seem to face more difficulties participating in the labour market than those without CMDs.

For WD trajectories among the CMD group, the proportions of women in the ‘Increasing medium’ and ‘Fluctuant high’ trajectories (around 60%) were higher than in the ‘Constant low’ group (50%). Alaie et al. 2023 also found that women had 1.7-times higher odds of belonging to the ‘Medium fluctuant’ trajectory group than in the ‘Low constant group’ [39]. This result among private sector employees corroborates previous findings that women generally have more WD [40] following a CMD diagnosis than men.

The ‘Constant low’ CMD trajectory group for both WD and unemployment had a higher proportion of individuals with college/university education than the other trajectory groups among those with CMD. This finding is comparable with previous research among individuals with CMD without distinction for employment sector [5], among non-manual workers in the private sector (trade and retail industry) [41], and among private sector employees with elevated depressive/anxiety symptoms [39]; these studies showed higher educational levels among those with ‘Constant low’ WD trajectories. The ‘Constant low’ CMD trajectory groups for WD and unemployment in our study also had higher educational levels than the ‘Constant low’ non-CMD trajectory group, emphasising the importance of education among individuals with CMDs. Those with a CMD diagnosis but higher educational level may have more control over their working hours and workload than those with lower educational level, reducing their risk of WD [5, 42]. Moreover, individuals in the ‘Constant low’ trajectory groups for either WD or unemployment may have experienced a delayed onset of symptoms of CMD, enabling them to complete their higher education. Consequently, despite their CMD diagnosis, they have a better prospect of avoiding marginalisation in the labour market than those with lower educational level. Around 42% and 70% of individuals with any mental disorder have their onset by age 14 and 25 years, respectively [43]. Given the increasing significance of obtaining a quality education for job opportunities, this could elucidate why individuals with CMDs frequently face challenges in sustaining employment during adulthood [15, 16].

Only 7% of those with CMD followed a ‘Fluctuant high’ WD trajectory. This group followed an anticipated pattern of an initial increase of WD following a CMD diagnosis at specialised healthcare and then gradual decrease of WD due to improvement of symptoms of CMD through effective management of the disorders. However, this group also had already high levels of WD before the CMD diagnosis. It is possible that symptoms of CMD among these individuals were milder during those years. Therefore, they did not require contact with specialised healthcare; rather, they were managed in the primary healthcare settings. As their symptoms worsened, they possibly got a diagnosis in specialised healthcare and were included in our incident CMD cohort. Additionally, the individuals belonging to this group had a much higher proportion of healthcare use for musculoskeletal or other somatic disorders during the year before cohort entry than the ‘Constant low’ group. Musculoskeletal disorders are the second leading cause of WD in Sweden, after mental disorders [3], and may have contributed to this already high level of WD among the individuals in the ‘Fluctuant high’ trajectory, even before their diagnosis of CMD in specialised healthcare. Similar to our ‘Fluctuant high’ WD trajectory group, Helgesson et al. (2018) previously reported a group of young individuals with CMDs (8.5% of the study population) who followed a trajectory of high levels of WD already before the CMD diagnosis (around 3 months in Y-3), increasing until Y + 3 (around 7.5 months) and then decreasing gradually to 6 months in Y + 6 [5]. This trajectory group also had somewhat similar sociodemographic and clinical characteristics to what we observed in our study.

For the outcome of unemployment, the ‘Constant low’ CMD trajectory group were more likely to live in big cities and do non-manual work than the other trajectory groups. Higher opportunities for employment in urban settings [44] and non-manual workers having around two times lower risk of unemployment than manual workers, even after controlling for age, educational level and living area [45], may explain these findings.

### Methodological considerations

The study has several strengths; data from high-quality nationwide registers [28–33] allowed us to form a population-based study cohort of all private sector employees and follow them for a long duration (three years before and six years after a CMD diagnosis) without any selective attrition. Due to the breadth and depth of the population-level data, we could also form a comparison group matching on key socio-demographic covariates, enabling us to interpret whether certain results may be CMD-specific. There are also several limitations in our study. First, the exploratory nature of our analyses limits any possibility to comment on causal inferences. Trajectory groups identified by group-based trajectory analysis are not fixed groups that exist in reality. Instead, these are data-driven identification of clusters of individuals who are expected to follow an approximate trajectory of an outcome over time [35]. The choice of the final model specifying the optimal number of trajectory groups is guided by researchers’ expectations based on theory and previous literature, subject-related knowledge, and statistical criteria [35]. Therefore, any association between such groups as the dependent factor and another variable as a predictor cannot be considered causal. Second, as we used data from specialised healthcare to form the exposure group (incident CMD diagnosis) and to create the clinical factors at baseline, we may have missed CMD cases with less severe morbidity. However, the inclusion of information on antidepressant use from both specialised and primary healthcare to define incident CMD may have minimised any bias from this source. Although indications for antidepressant use may vary (e.g. chronic pain), it is commonly prescribed for CMDs [46]. Third, most of the included factors did not show any association with the WD and unemployment trajectories among both the CMD and the non-CMD groups. Only ≤ 12% of the variability across the trajectory groups identified for WD and unemployment among the CMD and the non-CMD group were explained by the included socio-demographic, work-related and clinical factors. This finding suggests that there could be additional factors of importance that are associated with WD, e.g. lifestyle factors, psychosocial work environment factors, and severity of CMD [18, 47], which we could not include due to lack of data on these variables. Therefore, future studies should investigate what other factors can be influential in explaining the membership to trajectories of high or increasing WD and unemployment among those with a CMD diagnosis in the private sector.

### Conclusion

While the trajectories of WD and unemployment were constantly low before and after a diagnosis of CMD for most young private sector employees, some unfavourable trajectories at medium and high levels were also identified. Individuals were more likely to be women, with low education and with musculoskeletal disorders for belonging to such WD trajectories and more likely to be manual workers, have low education and living outside big cities for unemployment trajectories. Therefore, these specific vulnerable groups require special attention regarding their return-to-work process following a CMD diagnosis to improve on individual and societal consequences of WD and unemployment.

## Supplementary Information

Below is the link to the electronic supplementary material.

Supplementary file1 (PDF 557 KB)

Supplementary file2 (XLSX 58 KB)

## Data Availability

The data used in this study cannot be made publicly available due to privacy regulations. According to the General Data Protection Regulation, the Swedish law SFS 2018:218, the Swedish Data Protection Act, the Swedish Ethical Review Act, and the Public Access to Information and Secrecy Act, these types of sensitive data can only be made available for specific purposes, including research, that meets the criteria for access to this sort of sensitive and confidential data as determined by a legal review. Readers may contact Professor Kristina Alexanderson (kristina.alexanderson@ki.se) regarding data availability.

## References

[1] OECD (2012) Sick on the job? Myths and realities about mental health and work. Sweden: OECD Publishing

[2] OECD (2013) Youth in Sweden, mental ill-health and the transition into the labor market mental health and work. Sweden: OECD Publishing

[3] Swedish Social Insurance Agency. Social Insurance in Figures 2023. Sweden 2023 [15 Dec 2023]. Available from: https://www.forsakringskassan.se/download/18.73da25b81888fb1e89b97d/1695274193538/social-insurance-in-figures-2023.pdf

[4] Kaltenbrunner Bernitz B, Grees N, Jakobsson Randers M, Gerner U, Bergendorff S (2013) Young adults on disability benefits in 7 countries. Scand J Public Health 41(12):3–26 24077622 10.1177/1403494813496931

[5] Helgesson M, Tinghog P, Wang M, Rahman S, Saboonchi F, Mittendorfer-Rutz E (2018) Trajectories of work disability and unemployment among young adults with common mental disorders. BMC Public Health 18(1):1228 30400785 10.1186/s12889-018-6141-y PMC6219052

[6] Dalsgaard S, Thorsteinsson E, Trabjerg BB, Schullehner J, Plana-Ripoll O, Brikell I et al (2020) Incidence rates and cumulative incidences of the full spectrum of diagnosed mental disorders in childhood and adolescence. JAMA Psychiat 77(2):155–164 10.1001/jamapsychiatry.2019.3523 PMC6902162 31746968

[7] Silva SA, Silva SU, Ronca DB, Gonçalves VSS, Dutra ES, Carvalho KMB (2020) Common mental disorders prevalence in adolescents: a systematic review and meta-analyses. PLoS ONE 15(4):e0232007 32324835 10.1371/journal.pone.0232007 PMC7179924

[8] Kessler RC, Amminger GP, Aguilar-Gaxiola S, Alonso J, Lee S, Ustun TB (2007) Age of onset of mental disorders: a review of recent literature. Curr Opin Psychiatry 20(4):359–364 17551351 10.1097/YCO.0b013e32816ebc8c PMC1925038

[9] Essau CA, Lewinsohn PM, Lim JX, Ho MR, Rohde P (2018) Incidence, recurrence and comorbidity of anxiety disorders in four major developmental stages. J Affect Disord 228:248–253 29304469 10.1016/j.jad.2017.12.014 PMC5807150

[10] Johansson R, Carlbring P, Heedman Å, Paxling B, Andersson G (2013) Depression, anxiety and their comorbidity in the Swedish general population: point prevalence and the effect on health-related quality of life. PeerJ 1:e98 23862109 10.7717/peerj.98 PMC3709104

[11] Veldman K, Reijneveld SA, Ortiz JA, Verhulst FC, Bültmann U (2015) Mental health trajectories from childhood to young adulthood affect the educational and employment status of young adults: results from the TRAILS study. J Epidemiol Community Health 69(6):588–593 25667302 10.1136/jech-2014-204421

[12] Feeney A, North F, Head J, Canner R, Marmot M (1998) Socioeconomic and sex differentials in reason for sickness absence from the Whitehall II study. Occup Environ Med 55(2):91–98 9614392 10.1136/oem.55.2.91 PMC1757555

[13] Laaksonen M, Martikainen P, Rahkonen O, Lahelma E (2008) Explanations for gender differences in sickness absence: evidence from middle-aged municipal employees from Finland. Occup Environ Med 65(5):325 18252767 10.1136/oem.2007.033910

[14] Leinonen T, Pietiläinen O, Laaksonen M, Rahkonen O, Lahelma E, Martikainen P (2011) Occupational social class and disability retirement among municipal employees–the contribution of health behaviors and working conditions. Scand J Work Environ Health 37(6):464–472 21727991 10.5271/sjweh.3182

[15] Amin R, Mittendorfer-Rutz E, Björkenstam E, Virtanen M, Helgesson M, Gustafsson N et al (2023) Time period effects in work disability due to common mental disorders among young employees in Sweden—a register-based cohort study across occupational classes and employment sectors. Eur J Public Health 33(2):272–278 36869754 10.1093/eurpub/ckad026 PMC10066471

[16] Björkenstam E, Helgesson M, Gustafsson K, Virtanen M, Hanson LLM, Mittendorfer-Rutz E (2021) Sickness absence due to common mental disorders in young employees in Sweden: are there differences in occupational class and employment sector? Soc Psychiatry Psychiatr Epidemiol. 57(5): 1097–1106 10.1007/s00127-021-02152-3 PMC9042979 34386867

[17] Whittaker W, Sutton M, Macdonald S, Maxwell M, Smith M, Wilson P et al (2012) The effect of mental ill health on absence from work in different occupational classifications: analysis of routine data in the British household panel survey. J Occup Environ Med 54(12):1539–1544 23128300 10.1097/JOM.0b013e3182677d12

[18] Allebeck P, Mastekaasa A (2004) Swedish council on technology assessment in health care (SBU). Chapter 5. Risk factors for sick leave–general studies. Scand J Public Health Suppl. 63: 49–108 10.1080/14034950410021853 15513654

[19] Samuelsson A, Alexanderson K, Ropponen A, Lichtenstein P, Svedberg P (2012) Incidence of disability pension and associations with socio-demographic factors in a Swedish twin cohort. Soc Psychiatry Psychiatr Epidemiol. 47(12): 1999–2009. Swedish Public Employment Service (Arbetsförmedlingen). Perspektiv på långtidsarbetslösheten 2022: En deskriptiv analys (Perspective on unemployment 2022: a descriptive analysis. In Swedish) 2023 [12 Jan 2024]. Available from: https://arbetsformedlingen.se/download/18.e4c7c0717f2869663d1681b/1656493289843/perspektiv-pa-langtidsarbetslosheten-2022-en-deskriptiv-analys.pdf 10.1007/s00127-012-0498-5 22430867

[20] Swedish Public Employment Service (Arbetsförmedlingen). Perspektiv på långtidsarbetslösheten 2022: En deskriptiv analys (Perspective on unemployment 2022: a descriptive analysis. In Swedish) 2023 [12 Jan 2024]. Available from: https://arbetsformedlingen.se/download/18.e4c7c0717f2869663d1681b/1656493289843/perspektiv-pa-langtidsarbetslosheten-2022-en-deskriptiv-analys.pdf

[21] Swedish Public Employment Service (Arbetsförmedlingen). Vad förklarar den regionala arbetslösheten? En studie av vad demografin kan och inte kan förklara av den regionala arbetslösheten (What explains regional unemployment? A study of what demography can and cannot explain regional unemployment. In Swedish) 2023 [12 Jan 2024]. Available from: https://arbetsformedlingen.se/download/18.5f890e31185c874ca00198f/1676041122672/vad-forklarar-den-regionala-arbetslosheten-2023.pdf.Allebeck P, Mastekaasa A (2004) Swedish council on technology assessment in health care (SBU). Chapter 5. Risk factors for sick leave–general studies. Scand J Public Health Suppl. 63: 49–108 10.1080/14034950410021853 15513654

[22] Björkenstam E, Helgesson M, Gustafsson K, Virtanen M, Hanson LLM, Mittendorfer-Rutz E (2021) Occupational class and employment sector differences in common mental disorders: a longitudinal Swedish cohort study. Eur J Public Health 31(4):809–815 34269384 10.1093/eurpub/ckab091

[23] McGrath JJ, Lim CCW, Plana-Ripoll O, Holtz Y, Agerbo E, Momen NC et al (2020) Comorbidity within mental disorders: a comprehensive analysis based on 145 990 survey respondents from 27 countries. Epidemiol Psychiatr Sci 29:e153 32782057 10.1017/S2045796020000633 PMC7443806

[24] Stubbs B, Vancampfort D, Veronese N, Kahl KG, Mitchell AJ, Lin PY et al (2017) Depression and physical health multimorbidity: primary data and country-wide meta-analysis of population data from 190 593 people across 43 low- and middle-income countries. Psychol Med 47(12):2107–2117 28374652 10.1017/S0033291717000551

[25] Wallerblad A, Möller J, Forsell Y (2012) Care-seeking pattern among persons with depression and anxiety: a population-based study in Sweden. Int J Fam Med 2012(1):895425 10.1155/2012/895425 PMC3357962 22655197

[26] Mossialos E, Wenzl M, Osborn R, Sarnak D (Eds) (2016) International profiles of health care systems, 2015, the commonwealth fund

[27] Forslund T, Kosidou K, Wicks S, Dalman C (2020) Trends in psychiatric diagnoses, medications and psychological therapies in a large Swedish region: a population-based study. BMC Psychiatry 20(1):328 32576173 10.1186/s12888-020-02749-z PMC7313191

[28] Ludvigsson JF, Svedberg P, Olén O, Bruze G, Neovius M (2019) The longitudinal integrated database for health insurance and labour market studies (LISA) and its use in medical research. Eur J Epidemiol 34(4):423–437 30929112 10.1007/s10654-019-00511-8 PMC6451717

[29] Forsberg L, Rydh H, Björkenstam E, Jacobsson A, Nyqvist K, Heurgren M (1964) Kvalitet och innehåll i patientregistret. Utskrivningar från slutenvården. 2007: 1997–2007

[30] Ludvigsson JF, Andersson E, Ekbom A, Feychting M, Kim JL, Reuterwall C et al (2011) External review and validation of the Swedish national inpatient register. BMC Public Health 11:450 21658213 10.1186/1471-2458-11-450 PMC3142234

[31] Wettermark B, Hammar N, Fored CM, Leimanis A, Otterblad Olausson P, Bergman U et al (2007) The new Swedish prescribed drug register–opportunities for pharmacoepidemiological research and experience from the first six months. Pharmacoepidemiol Drug Saf 16(7):726–735 16897791 10.1002/pds.1294

[32] Social Insurance Agency (Försäkringskassan). MiDAS: Sjukpenning och Rehabiliteringspenning. (MiDAS: Sickness benefit and Rehabilitation benefit.) (In Swedish): Försäkringskassan (Social Insurance Agency); 2011 [09 Jan 2023]. Available from: https://www.forsakringskassan.se/download/18.5b8b0bec183b9d817dc11f/1666183936381/dokumentation-av-midas-sjukpenning-och-rehabiliteringspenning.pdf

[33] Brooke HL, Talbäck M, Hörnblad J, Johansson LA, Ludvigsson JF, Druid H et al (2017) The Swedish cause of death register. Eur J Epidemiol 32(9):765–773 28983736 10.1007/s10654-017-0316-1 PMC5662659

[34] Cote S, Tremblay RE, Nagin D, Zoccolillo M, Vitaro F (2002) The development of impulsivity, fearfulness, and helpfulness during childhood: patterns of consistency and change in the trajectories of boys and girls. J Child Psychol Psychiatry 43(5):609–618 12120857 10.1111/1469-7610.00050

[35] Jones BL, Nagin DS (2007) Advances in group-based trajectory modeling and an SAS procedure for estimating them. Soc Method Res 35(4):542–571

[36] Nagin DS, Odgers CL (2010) Group-based trajectory modeling in clinical research. Annu Rev Clin Psychol 6(1):109–138 20192788 10.1146/annurev.clinpsy.121208.131413

[37] van der Nest G, Lima Passos V, Candel MJJM, van Breukelen GJP (2020) An overview of mixture modelling for latent evolutions in longitudinal data: modelling approaches, fit statistics and software. Adv Life Course Res 43:100323 36726256 10.1016/j.alcr.2019.100323

[38] van de Schoot R, Sijbrandij M, Winter SD, Depaoli S, Vermunt JK (2017) The GRoLTS-checklist: guidelines for reporting on latent trajectory studies. Struct Equ Modeling 24(3):451–467

[39] Alaie I, Svedberg P, Ropponen A, Narusyte J (2023) Longitudinal trajectories of sickness absence among young adults with a history of depression and anxiety symptoms in Sweden. J Affect Disord 339:271–279 37437735 10.1016/j.jad.2023.07.014

[40] Olsson C, Tinnerholm Ljungberg H, Björk Brämberg E, Jensen I, Nybergh L (2021) A gender perspective on sick leave among young adults—barriers and resources for return to work as experienced by young employees and managers: a protocol for a qualitative study. Int J Qual Methods 20:16094069211032072

[41] Farrants K, Alexanderson K (2022) Trajectories of sickness absence and disability pension days among 189,321 white-collar workers in the trade and retail industry; a 7-year longitudinal Swedish cohort study. BMC Public Health 22(1):1592 35987617 10.1186/s12889-022-14005-y PMC9392931

[42] Hultin H, Hallqvist J, Alexanderson K, Johansson G, Lindholm C, Lundberg I et al (2010) Low level of adjustment latitude–a risk factor for sickness absence. Eur J Public Health 20(6):682–688 20142397 10.1093/eurpub/ckp240

[43] Solmi M, Radua J, Olivola M, Croce E, Soardo L, Salazar de PG, et al. (2022) Age at onset of mental disorders worldwide: large-scale meta-analysis of 192 epidemiological studies. Mol Psychiatry. 27(1): 281–95 10.1038/s41380-021-01161-7 PMC8960395 34079068

[44] Matthews R, Pendakur R, Young N (2009) Social capital, labour markets, and job-finding in urban and rural regions: comparing paths to employment in prosperous cities and stressed rural communities in Canada. Sociol Rev 57(2):306–330

[45] Lahtinen H, Sirniö O, Martikainen P (2020) Social class and the risk of unemployment: trends, gender differences and the contribution of education. Acta Sociol 63(3):303–321

[46] National Health Service (United Kingdom). Overview—antidepressants. 2023 [26 Mar 2024]. Available from: https://www.nhs.uk/mental-health/talking-therapies-medicine-treatments/medicines-and-psychiatry/antidepressants/overview/

[47] de Vries H, Fishta A, Weikert B, Rodriguez Sanchez A, Wegewitz U (2018) Determinants of sickness absence and return to work among employees with common mental disorders: a scoping review. J Occup Rehabil 28(3):393–417 28980107 10.1007/s10926-017-9730-1 PMC6096498

