# Supplementary material for: Trajectories of work disability and unemployment before and after a common mental disorder diagnosis among young private sector employees in Sweden–a register-based longitudinal study
Source: Soc Psychiatry Psychiatr Epidemiol. 2024 Oct 4;60(6):1421–35. doi: 10.1007/s00127-024-02777-0 (PMC12162682; doi:10.1007/s00127-024-02777-0)
Supplement: Supplementary file 1 — Supplementary file1 (PDF 557 KB) [file 127_2024_2777_MOESM1_ESM.pdf]

Supplementary information 1 for the article entitled *'Trajectories of work disability and unemployment before and after a common mental disorder diagnosis among young private sector employees in Sweden - a register-based longitudinal study'* submitted to the journal *'Social Psychiatry and Psychiatric Epidemiology'*.

Authors: Ridwanul Amin (PhD)[1,2], Emma Björkenstam (PhD)[1], Magnus Helgesson (PhD)[1,3], Ellenor-Mittendorfer-Rutz (PhD)[1]

[1]Division of Insurance Medicine, Department of Clinical Neuroscience, Karolinska Institutet, SE-171 77 Stockholm, Sweden

[2]Division of Infectious Diseases, Department of Medicine, Karolinska Institutet, SE-171 77 Stockholm, Sweden

[3]Department of Public Health and Caring Sciences, Public Health, Working Life and Rehabilitation, Uppsala University, SE-752 37, Uppsala, Sweden.

Corresponding author (email address): Ridwanul Amin (ridwanul.amin@ki.se)

List of figures

Supplementary Fig S1. Selection steps of the study population. 2
Supplementary figure S2a. Trajectory groups of work disability and unemployment, adjusted for educational level, three years before (Y-3) and six years after (Y+6) an incident (Y0) diagnosis of common mental disorder (CMD) in 2014 among young employees in the private sector, aged 22-29 years, gainfully employed and residing in Sweden on 31-Dec 2014 (n=12,121; exposure group) and the 1:1 matched (on age, sex, education and living area) comparison group without any incident CMD in 2014. 3
Supplementary figure S2b. Trajectory groups of work disability and unemployment, adjusted for other mental disorder, three years before (Y-3) and six years after (Y+6) an incident (Y0) diagnosis of common mental disorder (CMD) in 2014 among young employees in the private sector, aged 22-29 years, gainfully employed and residing in Sweden on 31-Dec 2014 (n=12,121; exposure group) and the 1:1 matched (on age, sex, education and living area) comparison group without any incident CMD in 2014. 4
Supplementary figure S2c. Trajectory groups of work disability and unemployment, adjusted for other somatic disorder, three years before (Y-3) and six years after (Y+6) an incident (Y0) diagnosis of common mental disorder (CMD) in 2014 among young employees in the private sector, aged 22-29 years, gainfully employed and residing in Sweden on 31-Dec 2014 (n=12,121; exposure group) and the 1:1 matched (on age, sex, education and living area) comparison group without any incident CMD in 2014. 5
Supplementary figure S3. Trajectory groups of work disability and unemployment three years before (Y-3) and seven years after (Y+6) an incident (Y0) diagnosis of common mental disorder (CMD) in 2014 among young employees in the private sector, aged 22-29 years, gainfully employed and residing in Sweden on 31-Dec 2014 who did not have any specialised healthcare for mental disorders during 2011-2014 (n=7498; exposure group) and the comparison group without any incident CMD in 2014 or any specialised healthcare for mental disorders during 2011-2014 (n=11,802). 6

List of tables

Supplementary table S4. List of Items of the Guidelines for Reporting on Latent Trajectory Studies (GRoLTS) Checklist: Guidelines for Reporting on Latent Trajectory Studies (1). 7
Supplementary table S5. Goodness of fit statistics* 9

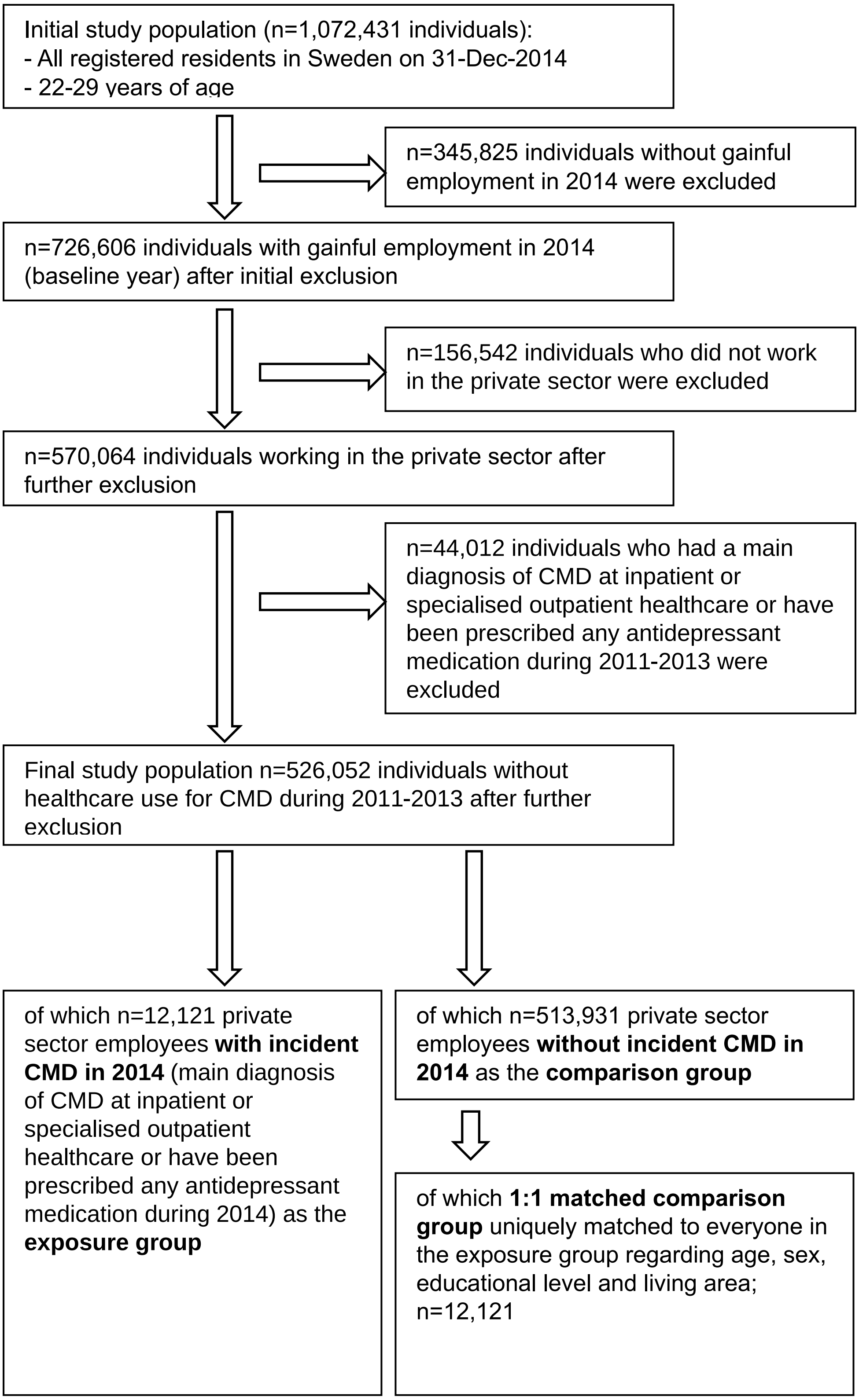

Supplementary Fig S1. Selection steps of the study population.

Supplementary figure S2a. Trajectory groups of work disability and unemployment, adjusted for educational level, three years before (Y-3) and six years after (Y+6) an incident (Y0) diagnosis of common mental disorder (CMD) in 2014 among young employees in the private sector, aged 22-29 years, gainfully employed and residing in Sweden on 31-Dec 2014 (n=12,121; exposure group) and the 1:1 matched (on age, sex, education and living area) comparison group without any incident CMD in 2014.

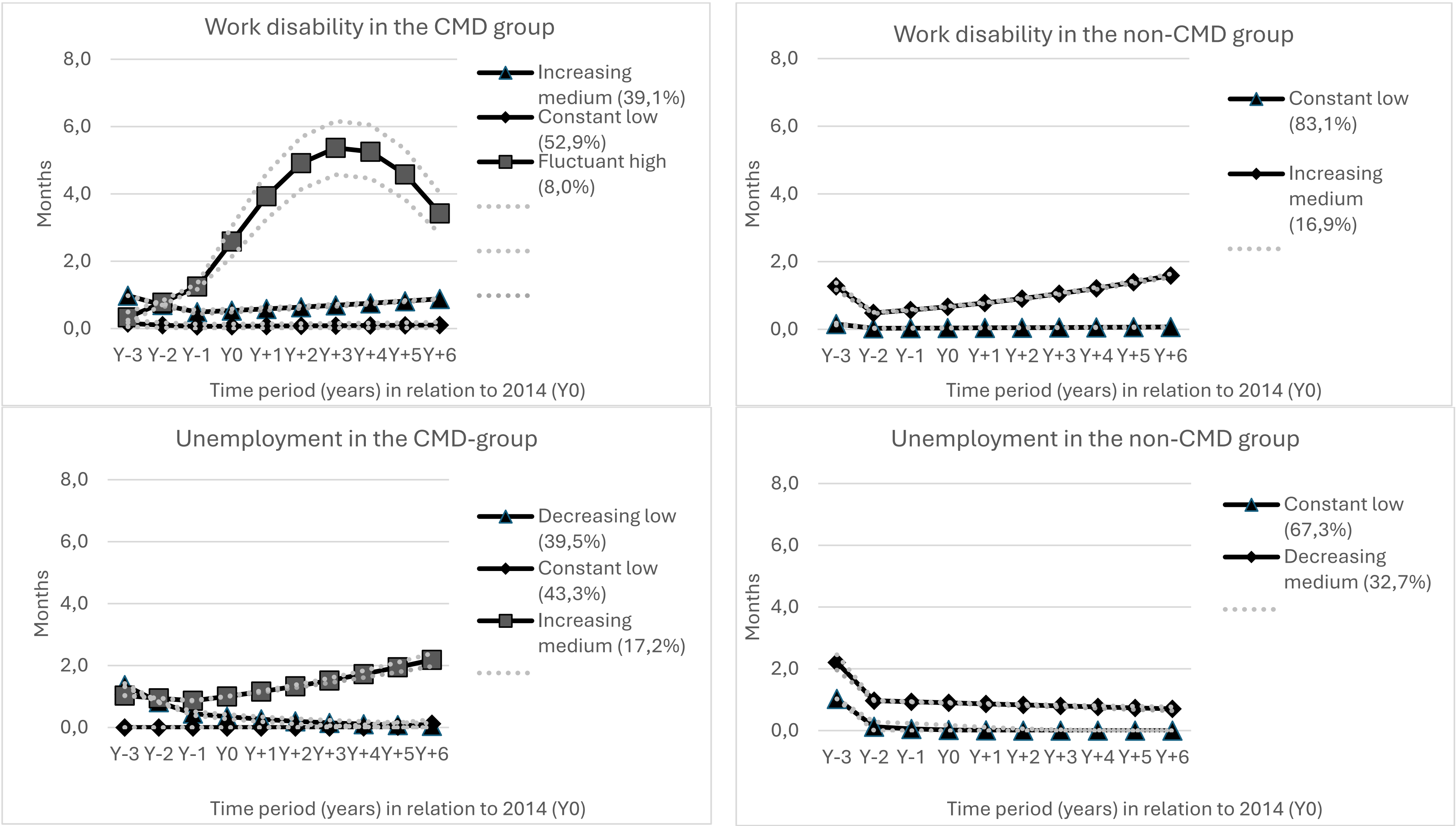

Supplementary figure S2b. Trajectory groups of work disability and unemployment, adjusted for other mental disorder, three years before (Y-3) and six years after (Y+6) an incident (Y0) diagnosis of common mental disorder (CMD) in 2014 among young employees in the private sector, aged 22-29 years, gainfully employed and residing in Sweden on 31-Dec 2014 (n=12,121; exposure group) and the 1:1 matched (on age, sex, education and living area) comparison group without any incident CMD in 2014.

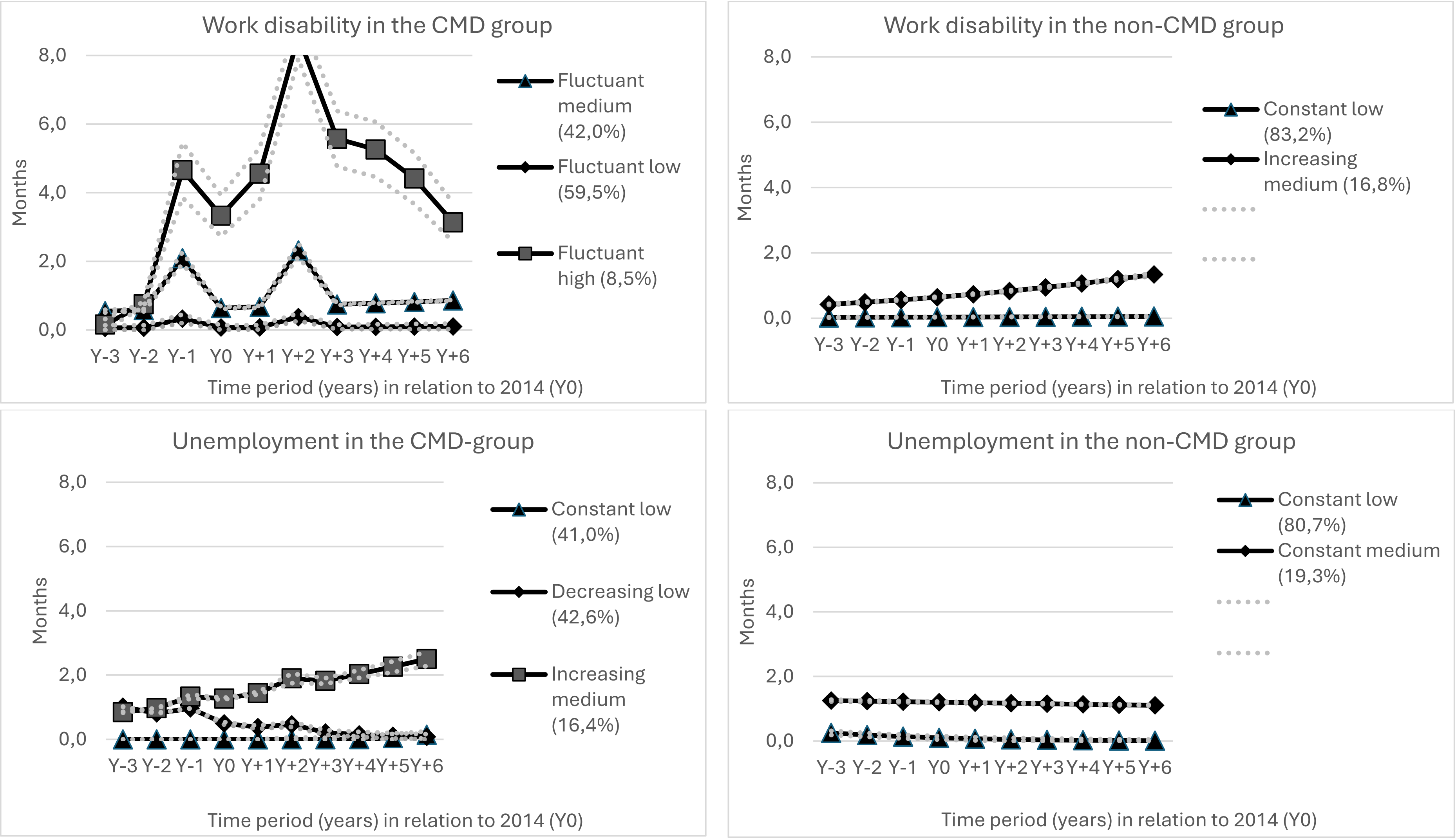

Supplementary figure S2c. Trajectory groups of work disability and unemployment, adjusted for other somatic disorder, three years before (Y-3) and six years after (Y+6) an incident (Y0) diagnosis of common mental disorder (CMD) in 2014 among young employees in the private sector, aged 22-29 years, gainfully employed and residing in Sweden on 31-Dec 2014 (n=12,121; exposure group) and the 1:1 matched (on age, sex, education and living area) comparison group without any incident CMD in 2014.

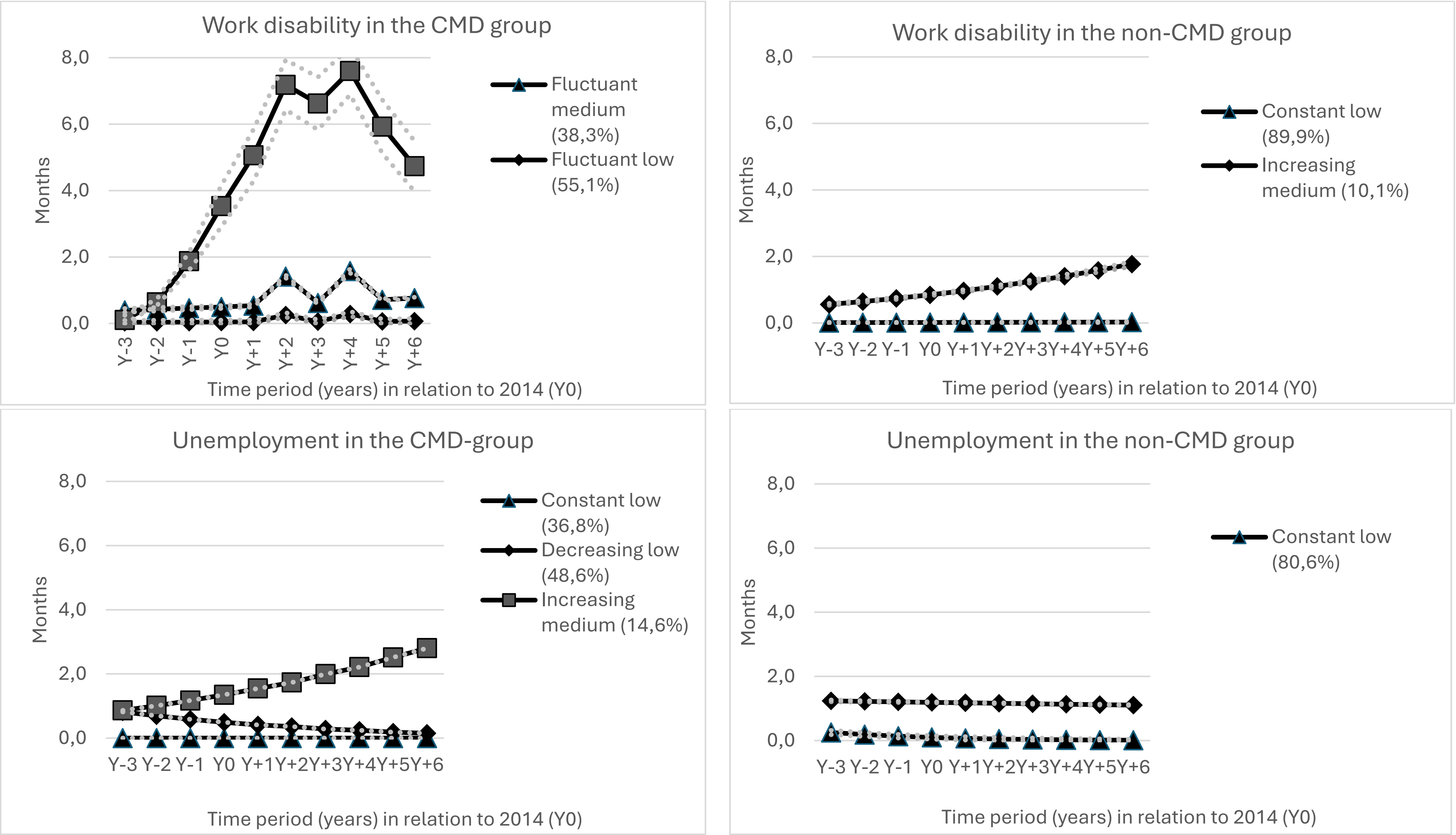

Supplementary figure S3. Trajectory groups of work disability and unemployment three years before (Y-3) and seven years after (Y+6) an incident (Y0) diagnosis of common mental disorder (CMD) in 2014 among young employees in the private sector, aged 22-29 years, gainfully employed and residing in Sweden on 31-Dec 2014 who did not have any specialised healthcare for mental disorders during 2011-2014 (n=7498; exposure group) and the comparison group without any incident CMD in 2014 or any specialised healthcare for mental disorders during 2011-2014 (n=11,802).

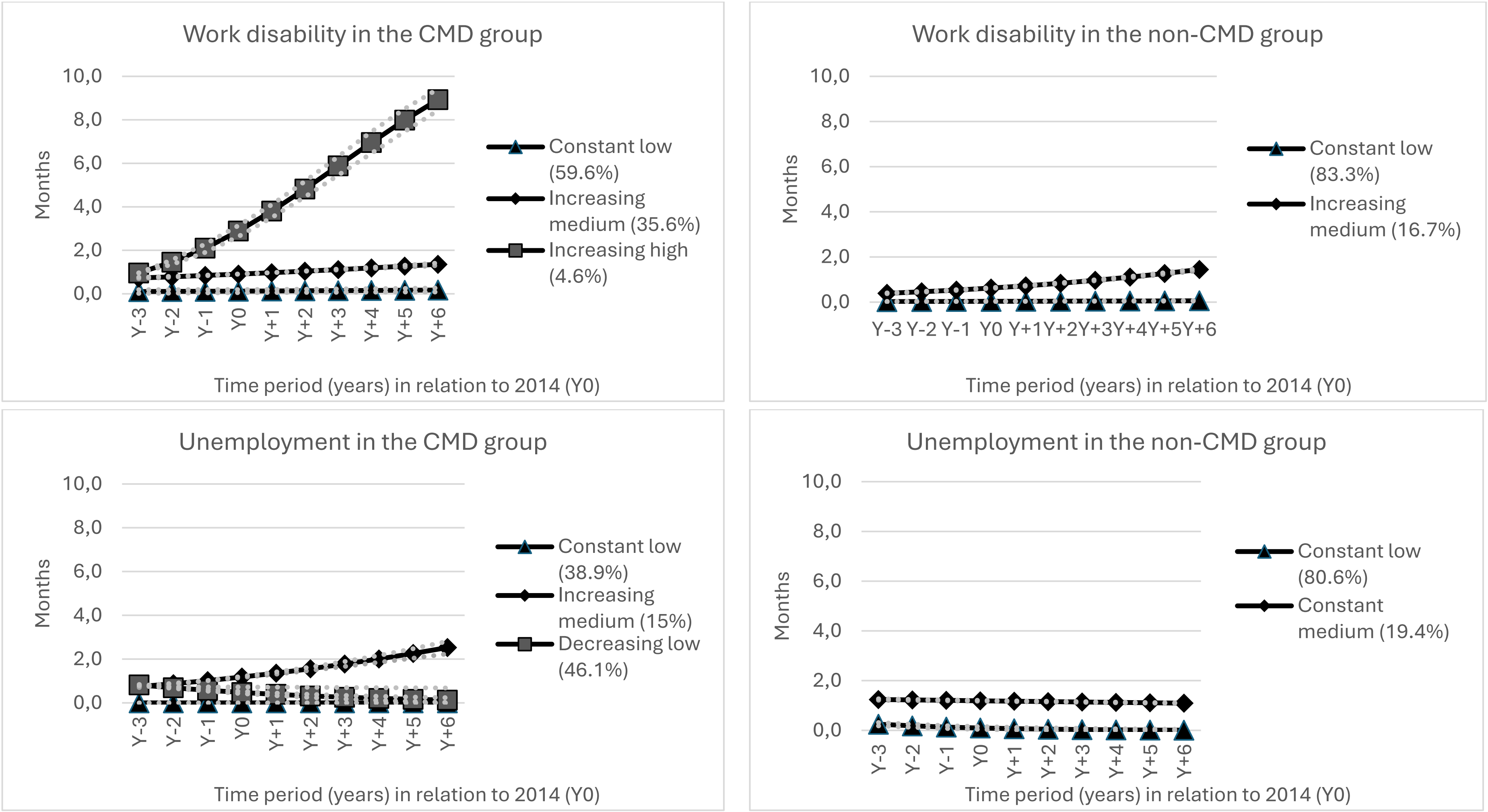

Supplementary table S4. List of Items of the Guidelines for Reporting on Latent Trajectory Studies (GRoLTS) Checklist: Guidelines for Reporting on Latent Trajectory Studies (1).

**Item**	**Note** (If reported, on which page/lines? If not reported, justification?)
1.Is the metric of time used in the statistical model reported?	Yes, reported on page 6, lines 25-29 and page 7, lines 6-7.
2.Is information presented about the mean and variance of time within a wave?	Not reported. The register-based data is time-structured and therefore, we consider minimal to no within-wave variability.
3a. Is the missing data mechanism reported?	Not reported. There is no missing data for the observed variables according to the definition of work disability and unemployment in the study. Drop-out due to death or emigration during a specific year of follow-up was considered as having missing outcome data for that year and the subsequent follow-up years. This is reported on page 8, lines 10-11.
3b. Is a description provided of what variables are related to attrition/missing data?	Not reported. Please see 3a.
3c. Is a description provided of how missing data in the analyses were dealt with?	Yes, please see 3a.
4.Is information about the distribution of the observed variables included?	Yes, reported on page 6, lines 25-29, page 6, lines 6-7 and Supplementary table S5. Due to the explorative nature of our study, we prioritized model convergence with censored-normal distribution (CNORM) over zero-inflated poisson distribution (ZIP) even if the data originally followed a ZIP distribution.
5.Is the software mentioned?	Yes, reported on page 7, lines 13-14.
6a. Are alternative specifications of within-class heterogeneity considered (e.g., LGCA vs. LGMM) and clearly documented? If not, was sufficient justification provided as to eliminate certain specifications from consideration?	Not reported. LGMM is computationally more demanding and often leads to model convergence issues. This justification is reported on page 7, lines 23-27.
6b. Are alternative specifications of the between-class differences in variance-covariance matrix structure considered and clearly documented? If not, was sufficient justification provided as to eliminate certain specifications from consideration?	Not reported. Previous literature suggested that variation in growth factors can be considered constant across subgroups and timepoints and therefore, this matrix was held fixed in this study. This justification is reported on page7, lines 26-27.

7.Are alternative shape/functional forms of the trajectories described?	Yes, on Supplementary table S5, we have reported the shapes of each of the trajectories in the final model.
8.If covariates have been used, can analyses still be replicated?	Yes, a standard three-step method was applied as described in the statistical analysis section. Please also see Supplementary figure S2a-S2c where covariates were adjusted in a one-step method.
9.Is information reported about the number of random start values and final iterations included?	Not reported since the statistical procedures converged to adequate results. Please see also the note on item 4 of this checklist.
10.Are the model comparison (and selection) tools described from a statistical perspective?	Yes, please see footnote in Supplementary table S4 on goodness of fit statistics.
11.Are the total number of fitted models reported, including a one-class solution?	Not reported, we only report statistics for the models selected from our criteria (please see Supplementary table S5.
12.Are the number of cases per class reported for each model (absolute sample size, or proportion)?	Yes, please see Fig. 1.
13.If classification of cases in a trajectory is the goal, is entropy reported?	Not reported. We think a single value of relative entropy does not provide sufficient information. The tables in supplementary information 2 and high average group probabilities suggest good adequacy of our models.
14a.Is a plot included with the estimated mean trajectories of the final solution?	Yes, please see Fig. 1.
14b.Are plots included with the estimated mean trajectories for each model?	No. Please see the note on item 4 of this checklist.
14c.Is a plot included of the combination of estimated means of the final model and the observed individual trajectories split out for each latent class?	Not included because even in the smallest trajectory group identified in this study, 7% of individuals with common mental disorders who followed a ‘fluctuant high’ work disability trajectory, such a plot is very busy and uninterpretable as the plot includes 851 observed individual trajectories.
15.Are characteristics of the final class solution numerically described (i.e., means, SD/SE, n, CI, etc.)?	Yes, please see Supplementary information 2.
16.Are the syntax files available (either in the appendix, supplementary materials, or from the authors)?	Yes, available from the authors on request.

Supplementary table S5. Goodness of fit statistics*

Trajectory model	Model	Order	BIC	AIC	Group	Average group probability
Work disability CMD group	CNORM	1 1 3	-106355.6	-106314.9	1	0.85
					2	0.88
					3	0.94
Work disability non-CMD group	ZIP	1 1	-47174.95	-47149.04	1	0.97
					2	0.93
Unemployment CMD group	CNORM	1 1 1	-82570.12	-82536.81	1	0.84
					2	0.92
					3	0.89
Unemployment non-CMD group	ZIP	1 1	-60293.15	-60270.94	1	0.96
					2	0.90

* We have tested models with 2, 3 and 4 trajectories but only the models that fulfilled the following criteria are presented: 1. The statistical procedures must converge to a solution with maximum likelihood estimates and have the best BIC and AIC values; 2. The average group probability must be > 0.70; 3. Each trajectory must consist of at least 5% of the observations.

References

1. van de Schoot R, Sijbrandij M, Winter SD, Depaoli S, Vermunt JK. The GRoLTS-Checklist: Guidelines for Reporting on Latent Trajectory Studies. Structural Equation Modeling: A Multidisciplinary Journal. 2017;24(3):451-67.
